# *OsLAP6/OsPKS1*, an orthologue of *Arabidopsis PKSA/LAP6*, is critical for proper pollen exine formation

**DOI:** 10.1186/s12284-017-0191-0

**Published:** 2017-12-28

**Authors:** Ting Zou, Qiao Xiao, Wenjie Li, Tao Luo, Guoqiang Yuan, Zhiyuan He, Mingxing Liu, Qiao Li, Peizhou Xu, Jun Zhu, Yueyang Liang, Qiming Deng, Shiquan Wang, Aiping Zheng, Lingxia Wang, Ping Li, Shuangcheng Li

**Affiliations:** 1 State Key Laboratory of Hybrid Rice, Rice Research Institute, Sichuan Agricultural University, Chengdu, 611130 China; 2 State Key Laboratory of Hybrid Rice, Hunan Hybrid Rice Research Center, Changsha, 410125 China; 3 Chongqing Key Laboratory of Economic Plant Biotechnology, Collaborative Innovation Center of Special Plant Industry in Chongqing, Institute of Special Plants, Chongqing University of Arts and Sciences, Yongchuan, 402160 China

**Keywords:** Rice, *OsLAP6/OsPKS1*, *PKSA/LAP6*, Male sterility, Pollen exine, Sporopollenin

## Abstract

**Background:**

Male fertility is crucial for rice yield, and the improvement of rice yield requires hybrid production that depends on male sterile lines. Although recent studies have revealed several important genes in male reproductive development, our understanding of the mechanisms of rice pollen development remains unclear.

**Results:**

We identified a rice mutant *oslap6* with complete male sterile phenotype caused by defects in pollen exine formation. By using the MutMap method, we found that a single nucleotide polymorphism (SNP) variation located in the second exon of *OsLAP6/OsPKS1* was responsible for the mutant phenotype. *OsLAP6/OsPKS1* is an orthologous gene of *Arabidopsis PKSA/LAP6*, which functions in sporopollenin metabolism. Several other loss-of-function mutants of *OsLAP6/OsPKS1* generated by the CRISPR/Cas9 genomic editing tool also exhibited the same phenotype of male sterility. Our cellular analysis suggested that *OsLAP6/OsPKS1* might regulate pollen exine formation by affecting bacula elongation. Expression examination indicated that *OsLAP6/OsPKS1* is specifically expressed in tapetum, and its product is localized to the endoplasmic reticulum (ER). Protein sequence analysis indicated that OsLAP6/OsPKS1 is conserved in land plants.

**Conclusions:**

*OsLAP6/OsPKS1* is a critical molecular switch for rice male fertility by participating in a conserved sporopollenin precursor biosynthetic pathway in land plants. Manipulation of *OsLAP6/OsPKS1* has potential for application in hybrid rice breeding.

**Electronic supplementary material:**

The online version of this article (doi: 10.1186/s12284-017-0191-0) contains supplementary material, which is available to authorized users.

## Background

Rice is one of the most significant crops in the world and is the staple food for nearly half of the global population (Virmani 1994; Cheng et al. 2007). Hybrid breeding strategy that relies on male sterile lines has been widely used to increase rice yield (Dar et al. 2013; Khush 2013). Therefore, pollen, as the male reproductive cell of rice, is closely associated with yield; and in-depth understanding of the mechanism of pollen development is extremely important for the improvement of rice yield.

The success of rice seeds formation depends on the production of vibrant pollen. As the protective structure of male gametes, pollen exine plays important roles in the development of pollen grains, resisting environmental stress, and the interaction of male and female gametes (Dumas et al. 1998; Hafidh et al. 2016; Mccormick 2004; Blackmore et al. 2007). The rice pollen exine, which is mainly composed of sporopollenin, comprises an outer layer (tectum), a foot layer (nexine), the middle bacula, and the tryphine in the cavities (Blackmore et al. 2007; Li and Zhang 2010; Zhang and Li 2014). The development of pollen exine involves three stages, including the formation and degradation of callose wall, the synthesis of primexine, and the secretion and deposition of sporopollenin (Godwin 1968; Rowley et al. 1981; Huang and Huang 2009). The biosynthesis of the sporopollenin precursors occurs in the tapetal cells, and these sporopollenin precursors are then transported to the surface of the microspore for exine formation (Domínguez et al. 1999; Liu and Fan 2013; Zhang et al. 2016). In general, the metabolic process of sporopollenin is critical for pollen exine development.

Over the past decade, an array of genes related to sporopollenin metabolism and pollen exine formation have been reported (Ariizumi and Toriyama 2010; Jiang et al. 2013; Shi et al. 2015). A T-DNA insertional mutant of rice *WDA1* gene showed significant defects in pollen exine formation (Jung et al. 2006). The acetylcholine A (acetyl-CoA) produced by the tapetal mitochondrial tricarboxylic acid cycle (TAC) is transported to plastid to form lauric acids under the catalytic action of WDAl (Jung et al. 2006). In the Arabidopsis tapetum, the medium-long-chain fatty acids generated from the plastids were modified by ACOS5 and transferred to the ER (de Azevedo Souza et al. 2009). Both mutants of *ACOS5* and its rice homologous gene *OsACOS12* had abnormal pollen exine development that resulted in male sterility (de Azevedo Souza et al. 2009; Yang et al. 2017; Zou et al. 2017a). In the ER, fatty acids are hydroxylated by two oxidases CYP703A and CYP704B (Morant et al. 2007; Yang et al. 2014; Dobritsa et al. 2009; Li et al. 2010). In the *cyp703a* and *cyp704b* mutants, the sporopollenin biosynthetic pathway was blocked, leading to smooth-surfaced pollen with the absence of the bacula and tectum formation (Li et al. 2010; Morant et al. 2007; Yang et al. 2014; Dobritsa et al. 2009; Yi et al. 2010). Furthermore, the pollen grains of the *Arabidopsis ms2* mutants showed flawed pollen exine. As a component of sporopollenin precursor in the form of fatty alcohols, the fatty acids hydroxylated by CYP703A or CYP704B are again acylated by ACOS5, and transported to the outside of ER under control of MS2 (Aarts et al. 1997; Chen et al. 2011; Wallace et al. 2015). At the same time, hydroxylated fatty acid can be catalyzed into phenolic substance, another component of sporopollenin precursor, by PKSA/LAP6 or PKSB/LAP5 (Dobritsa et al. 2010; Kim et al. 2010). The pollen grains in *pksa* or *pksb* single mutants are fertile but has abnormal pollen exine; however, their double mutants produced sterile pollen due to the significantly defective pollen exine (Dobritsa et al. 2010; Kim et al. 2010). In addition, the sporopollenin precursor components also include ultra-long-chain fatty acid derivatives, and FLPl were reported to be involved in this synthesis (Ariizumi and Toriyama 2010; Zhang et al. 2016; Ariizumi et al. 2003). The pollen generated by the *Arabidopsis flp1* mutant had an abnormal tryphine filling in its exine cavities, which resulted in a conditional male sterile phenotype and can be recovered by high humidity conditions (Ariizumi et al. 2003). Although much progress in sporopollenin metabolism of *Arabidopsis* pollen exine formation has been made recently, its mechanisms in rice is still ambiguous.

In this study, we characterized a complete male sterile mutant in the *indica* background, named *oslap6*, which produced aborted pollen with deformed pollen exine patterning. *OsLAP6* encodes an ortholog of *Arabidopsis* PKSA/LAP6 protein that participates in the pollen exine development by regulating sporopollenin metabolism. Knockout mutants of *OsLAP6* in the *japonica* background also exhibited pollen abortion. Expression examination showed that OsLAP6 protein is preferentially expressed in tapetum and is localized to the ER. OsLAP6 (also called OsPKS1) was previously reported to have similar enzymatic activity with PKSA/LAP6 (Wang et al. 2013). Our peptides alignment and phylogenetic analyses indicated that OsLAP6/OsPKS1 is conserved in land plants. Together with these results, we suggest that *OsLAP6/OsPKS1* is a key molecular switch of pollen exine formation in rice male reproductive development, and has possible applications in hybrid rice breeding.

## Results

### Characterization of the *oslap6* mutant

**Fig. 1:**
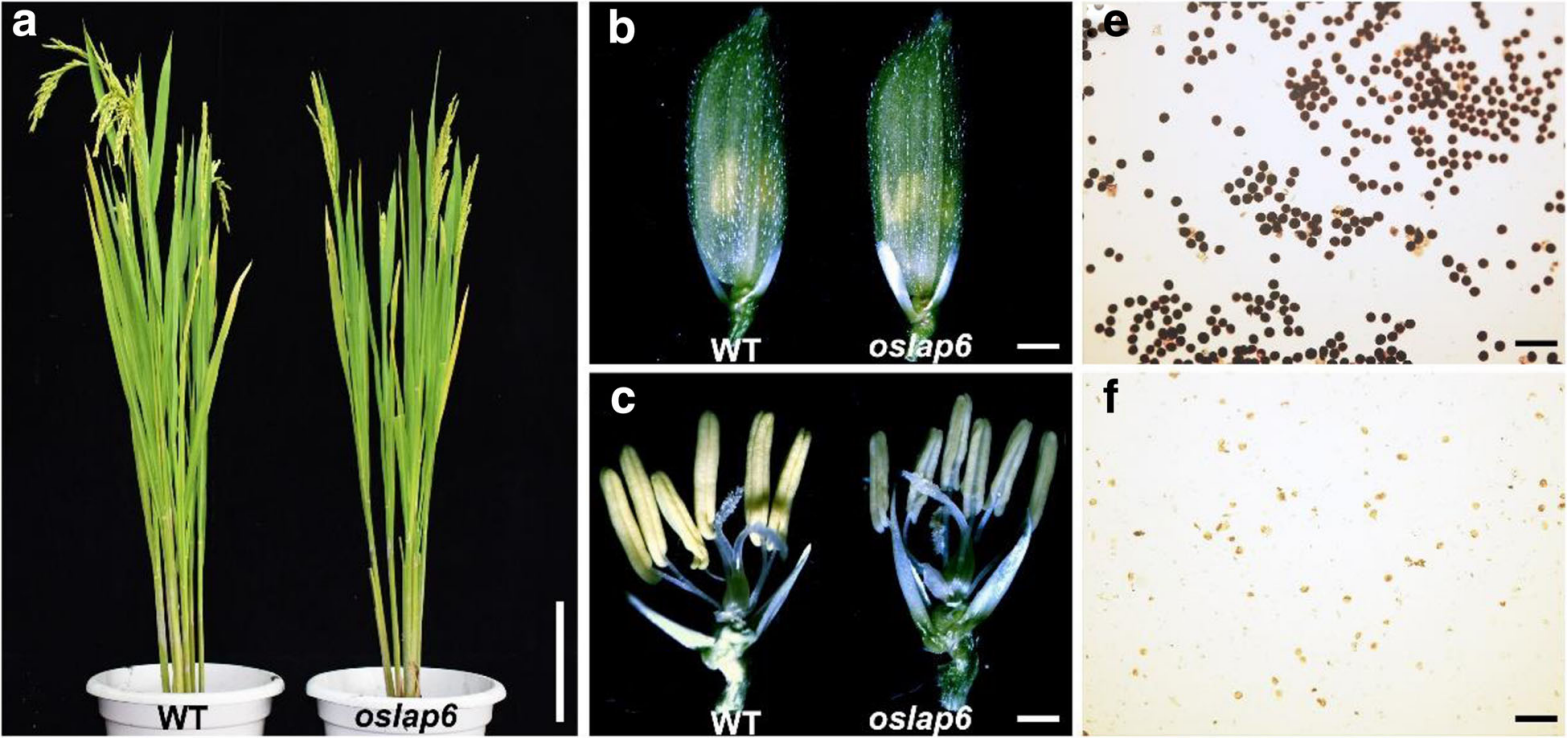
Phenotype of the WT and *oslap6.* **a** The WT and *oslap6* after heading. **b** Spikelets of the WT and *oslap6*. **c** Spikelets of the WT and *oslap6* in (**b**) with the palea and lemma removed. **d** and **e** Pollen grains of the WT and *oslap6* with I_2_ -KI staining, respectively. Scale bars = 20 cm (**a)**; 2 mm (**b**); 1 mm (**c**); 150 μm (**d** and **e**)

By screening the ethyl methanesulfonate (EMS)-induced rice mutant library of *indica* cultivar 9311, we characterized a male sterile mutant, named *oslap6*. The *oslap6* mutant displayed normally vegetative growth and floral development (Fig. 1a, b), but had smaller and pale yellow anthers during heading stage when compared with those of wild type 9311 (WT, Fig. 1c). Then we used I_2_-KI staining method to detect the pollen viability of *oslap6* mutant and WT. The results showed that all the pollen grains of *oslap6* were aborted (Fig. 1d, e), indicating that this mutant exhibited a complete male sterile phenotype. We backcrossed *oslap6* with the WT to generate F1 and F2 populations to investigate its genetic basis. All of the F1 plants were fertile as in the WT. In the F2 population, 146 fertile and 58 sterile plants were found, which agreed with a 3:1 segregation ratio (χ^2^ = 0.61 < χ^2^_0.05_ = 3.84) and indicated that a single recessive mutation controlled the male sterile phenotype of the *oslap6* mutant.

### Defects of pollen exine formation in *oslap6* mutant

**Fig. 2:**
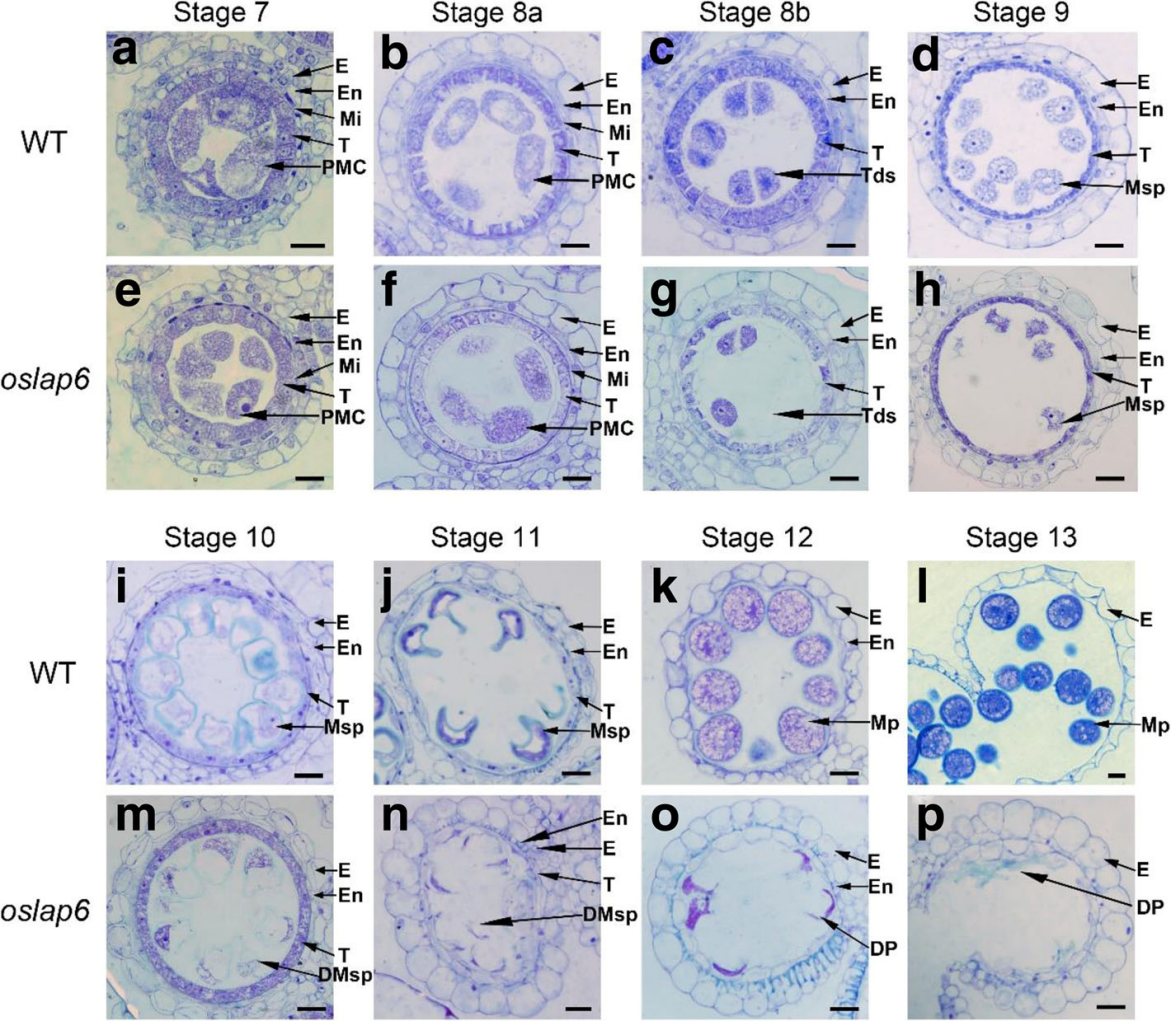
Transverse section comparison of anther development between the WT and *oslap6*. Eight stages of anther development between the WT and *oslap6* were compared. **a** and **e** Stage 7; **b** and **f** Stage 8a; **c** and **g** Stage 8b; **d** and **h** Stage 9; **i** and **m** Stage 10; **j** and **n** Stage 11; **k** and **o** Stage 12; **l** and **p** Stage 13; the WT sections are shown in **a**-**d** and **i**-**l**; Sections of the *oslap6* are shown in **e**-**h** and **m**-**p**. DMsp, degenerated microspore; DP, degenerated pollen; E, epidermis; En, endothecium; Mi, middle layer; MP, mature pollen; Msp, microspore; PMC, pollen mother cell; Tds, tetrads; T, tapetum. Scale bars =15 μm

To investigate the cytological mechanisms responsible for the pollen abortion of *oslap6* mutant, we performed transverse section analysis for the anthers of *oslap6* and WT. Recent studies classified the rice anther and pollen development into 14 stages (Zhang and Wilson 2009; Zhang et al. 2011). No obvious defects were found between WT (Fig. 2a-c) and *ospk1–1* anther (Fig. 2e-g) either in the formation of tetrads or in the development of the four-layer anther wall (the epidermis, endothecium, middle layer, and tapetum) until stage 8b. However, at stage 9, significant abnormalities of the microspore morphology were observed after the microspores were released from the tetrads. At this stage, the microspores were round shaped in the WT (Fig. 2d), while the *oslap6* microspores showed slight shrinkage (Fig. 2h). At stage 10, microspores with obvious pollen exine underwent vacuolization and enlarged in the WT (Fig. 2i). Although the *oslap6* microspores also exhibited the thin and weakly stained pollen exine, the contraction of microspore became more obvious at this stage (Fig. 2m). From stage 11 to stage 13, after two steps of mitosis division, the completion of pollen wall formation, and the accumulation of starch granules (Zhang et al. 2011), the WT microspores eventually developed into mature pollen (Fig. 2j-l). In contrast, the microspores of *oslap6* were irregularly developed with an abnormal shape of collapse; and ultimately formed aborted and adhesive pollen at stages 13 (Fig. 2n-p).

**Fig. 3:**
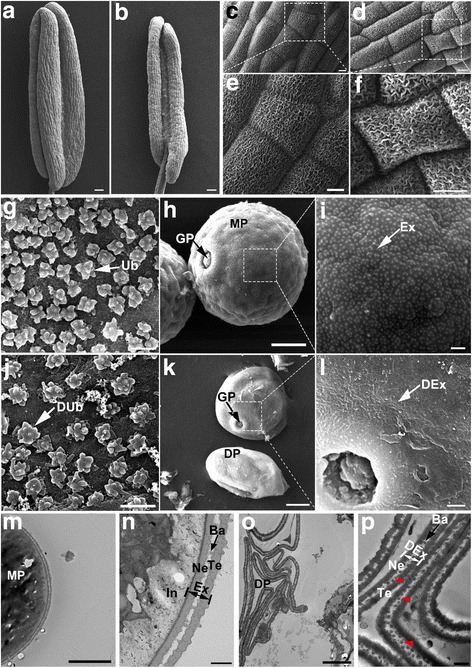
SEM and TEM observation for the WT and *oslap6* anther and pollen. **a**-**l** SEM analysis of the surfaces of anthers and pollen grains in the WT and *oslap6* at stage 12. **m**-**p** TEM observation of pollen exine in the WT and *oslap6* at stage 13. **a** and **b** Anthers of WT and *oslap6*. **c** and **e** Anther epidermis of WT and *oslap6*. **d** and **f** The enlarged images of epidermal surface of the WT and *oslap6* anthers. **g** and **j** The inner surface of the WT and *oslap6* anthers. **h** and **k** Pollen grains in the WT and *oslap6* anthers. **i** and **l** Outer surface of pollen grains in the WT and *oslap6* anthers. **m** and **o** Ultra-thin sections of pollen in the WT and *oslap6*. **n** and **p** The magnified images of pollen exine in the WT and *oslap6*, the arrows indicate the collapsed bacula. Ba, bacula; DEx, deformed exine; DP, degenerated pollen; DUb, deformed Ubisch body; Ex, exine; In, intine; GP, germination pore; MP, mature pollen; Ne, nexine; Te, tectum; Ub, Ubisch body; Scale bars = 100 μm (**a** and **b**); 10 μm (**c**–**f**, **h**, and **k**); 1 μm (**g**, **j**, **i**, and **l**); 2 μm (**m**); 5 μm (**o**); 500 nm (**n** and **p**)

To observe the defects of *oslap6* in more detail, we examined the anther samples of WT and *oslap6* at stage 12 by using scanning electron microscopy (SEM, Fig. 3). In agreement with the phenotypic observation results (Fig. 1c), the anthers of *oslap6* (Fig. 3b) were smaller than WT (Fig. 3a). Both the WT (Fig. 3c, e) and *oslap6* anther (Fig. 3d, f) showed well-formed cuticle on their surface. However, unlike the regular patterning of the Ubisch bodies arranged on the inner surface of WT anther locule (Fig. 3g), the *oslap6* had relatively less-organized Ubisch bodies with abnormal form (Fig. 3j). Consistent with the results of transverse section analysis (Fig. 2o), the shrinking and irregularly shaped pollen with smooth exine were observed in the *oslap6* anther (Fig. 3k, l), while the WT pollen grains were spherical and covered by pollen exine with intensively distributed spots (Fig. 3h, i). We also used transmission electron microscopy (TEM) to observe the pollen of WT and *oslap6* at stage 13. The results showed that, compared with the globular WT pollen grains (Fig. 3m) with normally structural exine (Fig. 3n), the pollen of *oslap6* were adhesive and aborted (Fig. 3o) with deformed exine with collapsed bacula (Fig. 3p). These results indicated that the defects of pollen exine formation led to the pollen abortion of the *oslap6* mutant, suggesting abnormal sporopollenin deposition.

### Cloning of *OsLAP6*

**Fig. 4:**
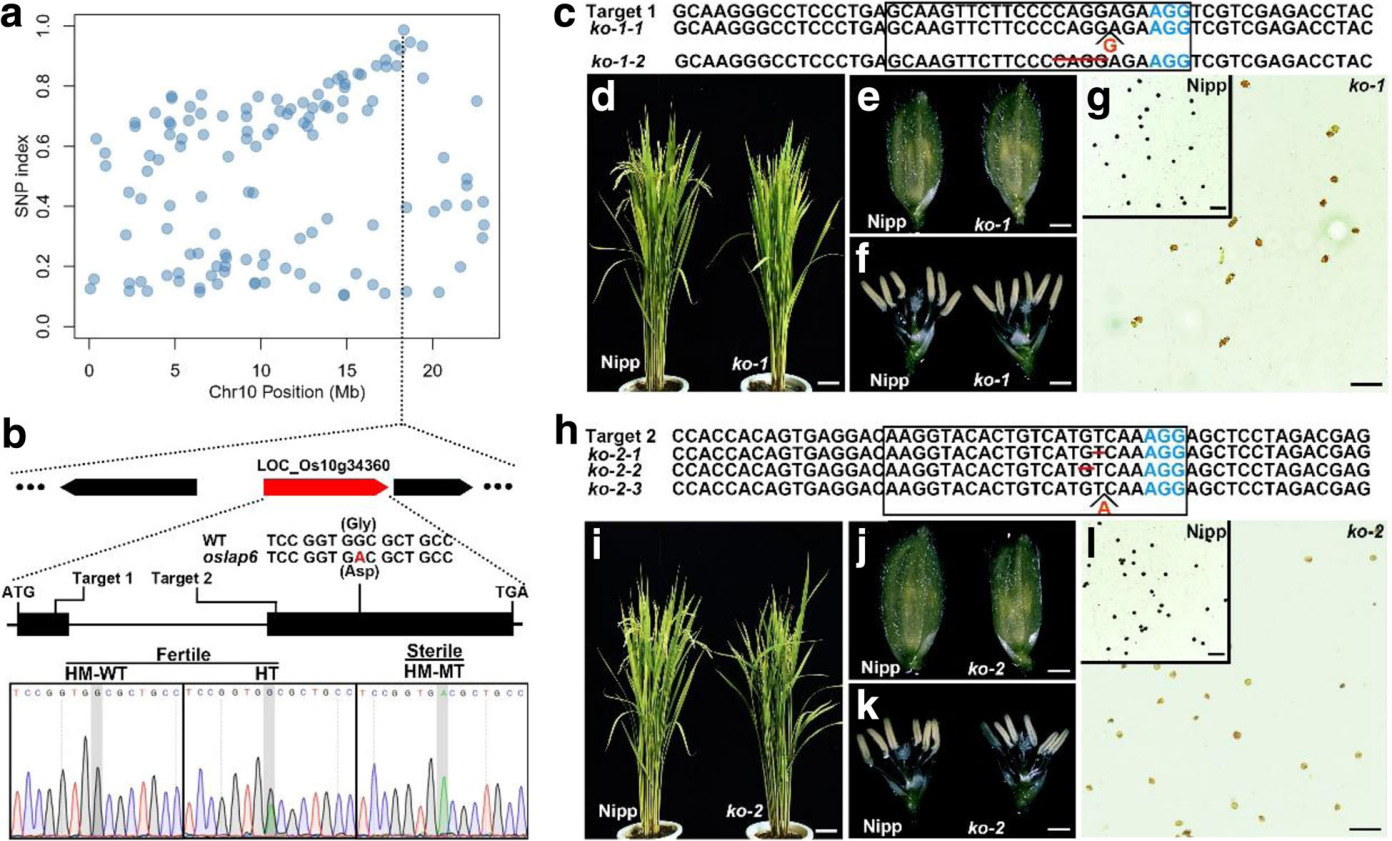
Molecular detection of *OsLAP6* and phenotypes of loss-of-function mutants. **a** The distribution of the SNP sites on chromosome 10. **b** *OsLAP6* gene structure, the mutation site of *oslap6* mutant, two independent sgRNA targeting sites of CRISPR/Cas9 system, and the identification of the *OsLAP6* gene in the *oslap6/9311* F2 population by sequencing. The black boxes indicate the exons. The red characters indicate the SNP mutation in *OsLAP6* gene. **c** and **h** Sequence alignment of the homologous mutants within the target1 and target2 in the *ko-1* and *ko-2* T_0_ plants, respectively. The black frames indicate the target sequences; and the PAM sequences and mutations are in blue and red, respectively. **d-g** Phenotype of WT and *ko-1* in the Nipponbare (Nipp) background. **i**-**l** Phenotype of WT and *ko-2* in the Nipp background. **d** and **i** Plants after heading. **e** and **j** Spikelets. **f** and **k** Spikelets with the palea and lemma removed. **g** and **l** Pollen grains with I_2_ -KI staining. Scale bars = 5 cm (**d** and **i)**; 1.5 mm (**e** and **j**); 1 mm (**f** and **k**); 150 μm (**g** and **l**)

We used MutMap cloning approach based on the next generation sequencing to identify the mutation that was responsible for male sterility in the *oslap6* mutant (Abe et al. 2012; Takagi et al. 2015). Our analysis revealed a region with a high SNP-index cluster between 17.79 Mbp and 18.69 Mbp on chromosome 10 (Fig. 4a and Additional file 1: Figure S1). In this region, we found a SNP variation at nucleotide position 18,317,741 (G to A) located on the second exon of *LOC_Os10g34360*. This mutation changed the 173rd glycine (Gly) into aspartic acid (Asp, Fig. 4b and Additional file 1: Figure S2). *LOC_Os10g34360* was annotated to encode a putative stilbene synthase (Rice Genome Annotation Project, http://rice.plantbiology.msu.edu/index.shtml), which showed a 61% identity with *Arabidopsis* PSKB/LAP6 (Additional file 1: Figure S3) that was involved in pollen exine development by regulating sporopollenin metabolism (Dobritsa et al. 2010; Kim et al. 2010). Thus, we speculated this point mutation within *LOC_Os10g34360* is responsible for the male sterile phenotype of *oslap6*.

To verify the association between the mutation and the male sterility of *oslap6*, we randomly selected 12 fertile plants and 48 sterile plants in the *oslap6/9311* F2 population for PCR amplification and sequencing. The results showed that all of the male sterile plants had homozygous mutations at this locus, whereas the fertile plants were homozygous wild type or heterozygous genotype (Fig. 4b and Table 1), indicating this mutation was co-segregated with the male sterile phenotype of *oslap6*, also suggesting that *LOC_Os10g34360* plays an essential role in rice male reproductive development.

**Table 1:** Association analysis of the genotype and phenotype of *oslap6* F2 plants

Phenotype	No. of plants examined	No. of plants with HM-MT *	No. of plants with HT **	No. of plants with HM-WT ***
Fertility	12	0	7	5
Male sterility	48	48	0	0

All these plants are in *indica* 9311 background. *, **, and *** indicate homozygous mutation, heterozygous genotype, and homozygous wild type genotype, respectively. No.,numbers

### Loss-of-function mutants of *OsLAP6* were also complete male sterile

To confirm the role of *LOC_Os10g34360* in rice male fertility, we designed two independent targets within this gene (Fig. 4b), and used the CRISPR/Cas9 genome editing system to investigate its function. A number of transgenic plants that may have mutations in target 1/2 in Nipponbare background were obtained. Direct or cloned sequencing of targeted regions showed that, among those T_0_ plants, the total mutation rate of these two target sites reached 81.5% (Table 2), and two and three independent homozygous mutants with different mutation types were found at target 1 and target 2, respectively (Fig. 4c, h). The protein sequence analysis predicted that all five mutations resulted in premature stop codons, and produced truncated polypeptides of this protein (Additional file 1: Figure S2), indicating the successful knocking out of *LOC_Os10g34360* within these homozygous mutants (loss-of-function mutants).

**Table 2:** Information of T_0_ plants found with CRISPR/Cas9-generated mutations in the target sequences

Target	No. of plants detected	No. of plants with mutations	Mutation rate (%)	No. of plants with HM-MT *	No. of plants with HT **	No. of plants with Ba-MT ***
1	22	19	86.4	2	5	12
2	30	23	76.7	3	9	11
Total	52	42	81.5	5	14	23

All these plants are in *japonica* Nipponbare background. *, **, and *** indicate homozygous mutations, heterozygous mutations, and biallelic mutations, respectively. No.,numbers

Subsequently, we used these loss-of-function mutants for phenotypic analysis. No detectable differences were found in vegetative growth or floral organ development between Nipponbare and loss-of-function mutants, but the pollen grains of all loss-of-function mutants were aborted (Fig. 4d-g and i-l). This result was consistent with the *oslap6* mutants (Fig. 1). Moreover, segregation analysis further showed that the plants with homozygous or biallelic mutations in the F2 progenies had complete male sterile phenotype, while the genotypes of fertile plants were homozygous wild type or heterozygous (Additional file 2: Table S1). These results indicated that loss-of-function of *LOC_Os10g34360* can result in complete male sterility, and *LOC_Os10g34360* is *OsLAP6*.

### Expression pattern of *OsLAP6*/*OsPKS1*

**Fig. 5:**
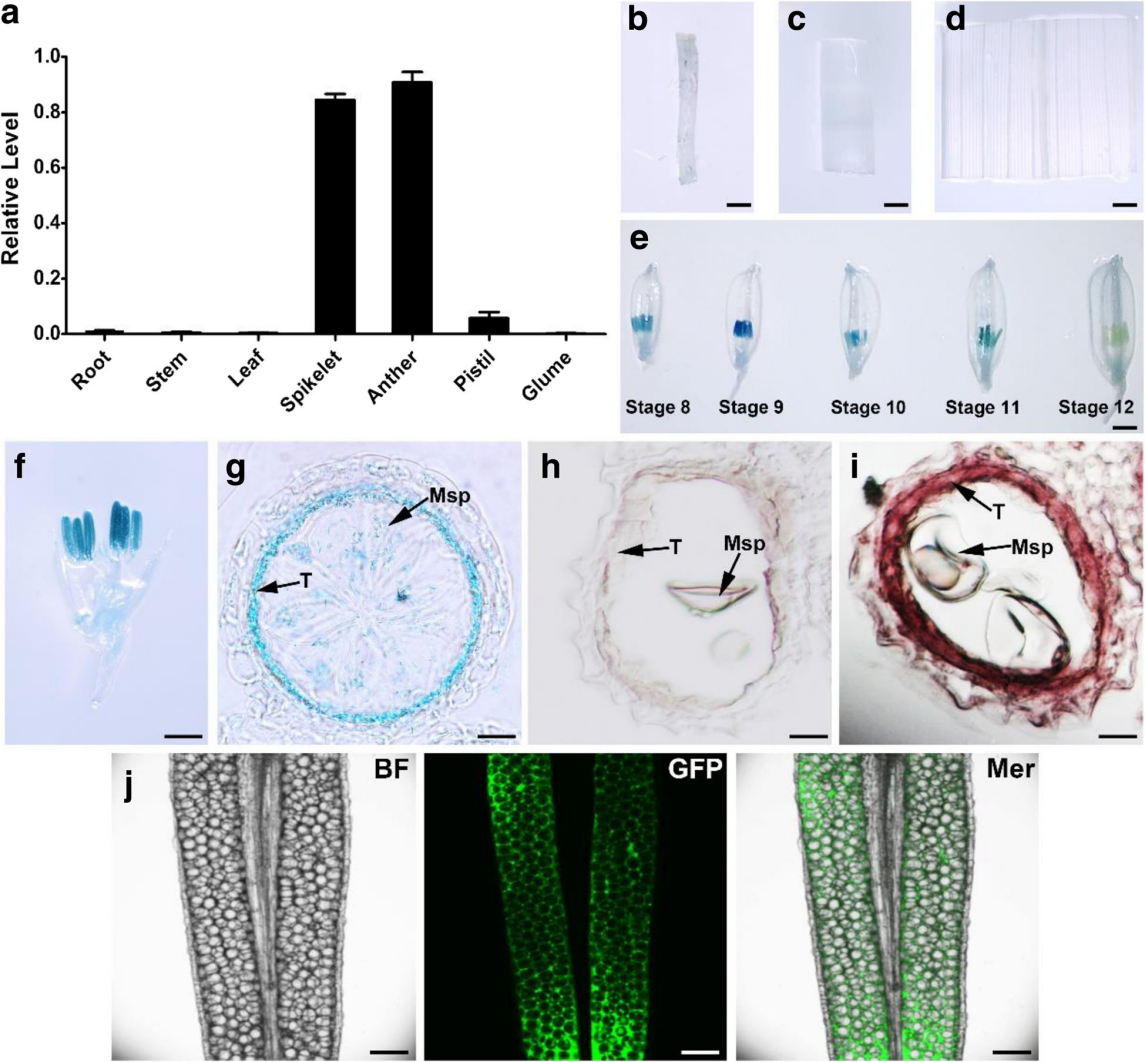
Expression pattern of *OsLAP6/OsPKS1*. **a** Expression analysis of *OsLAP6/OsPKS1* by qPCR. **b**-**g** GUS expression (blue staining) patterns of root, stem, leaf, spikelet at different anther developing stages, spikelet with the palea and lemma removed at stage 9, and the section of GUS-staining anther of the OsLAP6/OsPKS1_pro_::GUS transgenic line, respectively. **h** and **i** RNA in situ hybridization of *OsLAP6/OsPKS1* sense and antisense probe with the WT (Nipponbare) sections, respectively. **j** Confocal images of the hand free anther samples of OsLAP6/OsPKS1_pro_::OsLAP6/OsPKS1-GFP transgenic line. BF, bright field; GFP, green fluorescent protein channel; Mer, merged image of each channel. Msp, microspore; T, tapetum. Scale bars = 1 mm (**b**-**g**); 20 μm (**h**-**i**); 50 μm (**j**)

*OsLAP6* was also called *OsPKS1* (Wang et al. 2013). Our results showed that mutations of *OsLAP6/OsPKS1* only led to pollen abortion without affecting the development of vegetative organs, suggesting that *OsLAP6*/*OsPKS1* might be highly expressed in anther. Thus, we performed quantitative Real Time-PCR (qPCR) analysis to detect the expression level of *OsLAP6*/*OsPKS1* in a series of rice tissues, including root, stem, leaf, spikelet, anther, pistil, and glume. The results indicated that *OsLAP6*/*OsPKS1* was strongly expressed in spikelet, and was predominantly in anther (Fig. 5a). To confirm this temporal-spatial expression of *OsLAP6*/*OsPKS1*, we fused the native promoter of *OsLAP6*/*OsPKS1* with the β-glucuronidase (GUS) reporter gene and transformed it into Nipponbare. The GUS staining results showed that the expression of *OsLAP6/OsPKS1* was only detectable in developing anthers (Fig. b-f); and the maximal expression was observed within the anthers at stage 9 (Fig. 5e). Moreover, we examined the sections of GUS-staining anther. The results showed that GUS was highly stained in tapetum (Fig. 5g). We further analyzed the Nipponbare anther sections by RNA in situ hybridization (Fig. 5h and i); and notable hybridization signals were also detected in the tapetal cells (Fig. 5i). These results indicated that *OsLAP6/OsPKS1* gene is exclusively transcribed in tapetum.

To determine whether the OsLAP6/OsPKS1 protein is also expressed specifically in tapetal cells, we fused the green fluorescent protein (GFP) to the C-terminal of OsLAP6/OsPKS1, and the *OsLAP6/OsPKS1* native promoter drove this fusion protein, then we transformed this construct into Nipponbare. The results of confocal microscopy showed that OsLAP6/OsPKS1 was indeed expressed only in the tapetum (Fig. 5j). Based on these results, we conclude that *OsLAP6/OsPKS1* is not only specifically transcribed in the anther but is also preferentially expressed in the tapetum during male gametes development, which agreed with its important role in pollen development.

### Subcellular location of OsLAP6/OsPKS1 protein

**Fig. 6:**
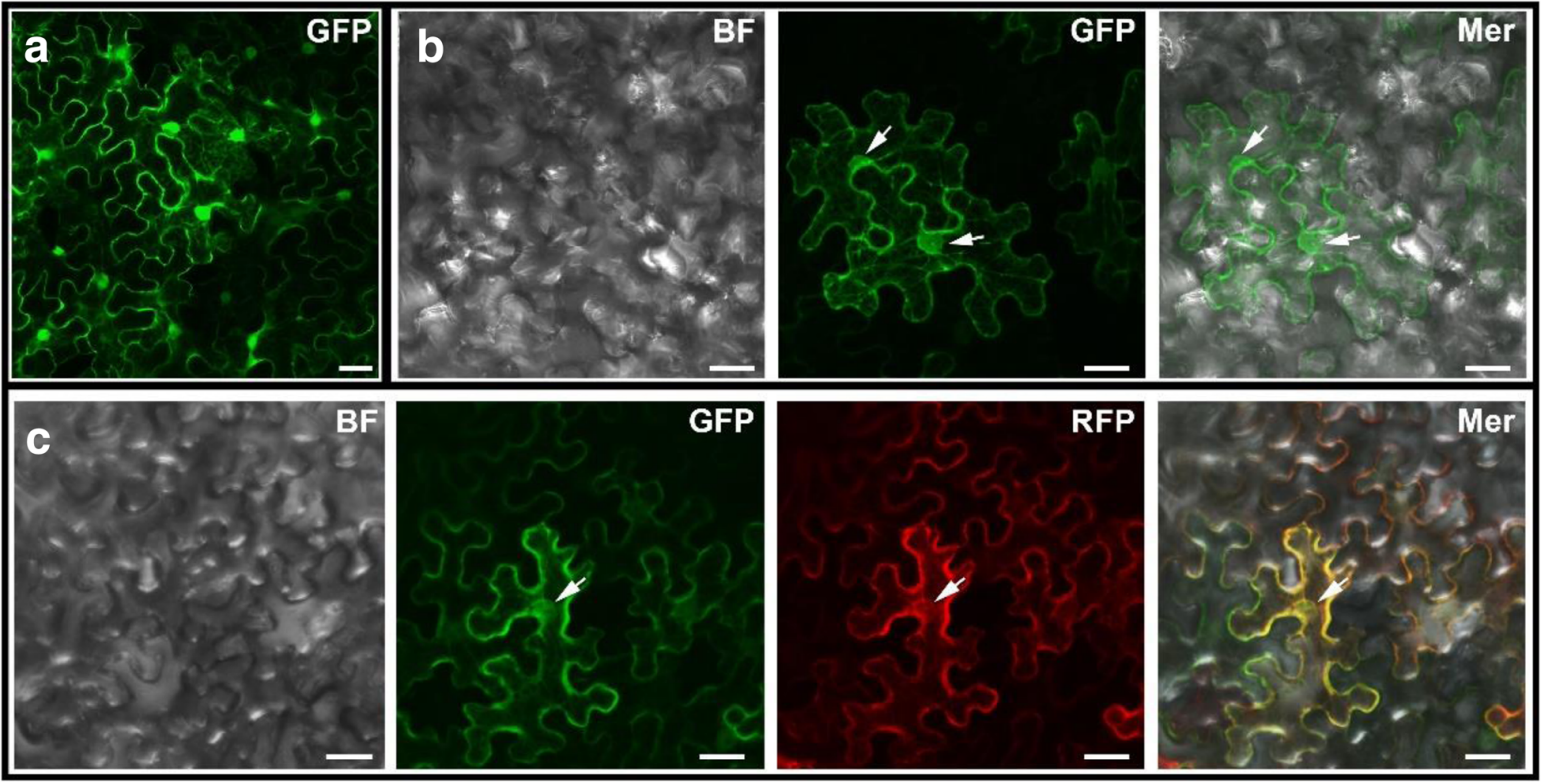
Subcellular Localization of OsLAP6/OsPKS1 in tobacco leaf epidermal cells. Confocal images of tobacco leaf epidermal cells after 72 h of infection were shown. **a** Transient expression of control, showing that the expression of the GFP protein was distributed throughout the cell. **b** Transient expression of OsLAP6/OsPKS1-GFP, showing that OsLAP6/OsPKS1 may localize to the ER-like structures. **c** Co-expression of OsLAP6/OsPKS1-GFP and ER-marker, showing the GFP signals of OsLAP6/OsPKS1-GFP are well merged with the RFP signals of ER-marker. The white arrows indicate the ER-ring. BF, bright field; GFP, green fluorescent protein channel; Mer, merged image of each channel. Scale bars = 20 μm

*Arabidopsis* PKSA/LAP6, an orthologous protein of OsLAP6/OsPKS1, was reported to participate in the sporopollenin metabolon localized to the ER in tapetal cells (Lallemand et al. 2013). Given the homology of these two proteins and the similar defects of pollen exine development in the *oslap6* and *pksb/lap6* mutants, we therefore predicted that OsLAP6/OsPKS1 protein was also localized to the ER. To verify this, we constructed the OsLAP6/OsPKS1-GFP under control of the double 35S promoter, and transiently expressed this plasmid in the epidermal cells of *Nicotiana benthamiana* (tobacco) leaves. Confocal microscopy results showed that the GFP signals of OsLAP6/OsPKS1-GFP were mainly observed on the ER-like structures (Fig. 6b). Thus, we co-transformed the OsLAP6/OsPKS1-GFP with the ER-marker, a red fluorescent protein (RFP) that fused with the KDEL ER-retention signal and also driven by the double 35S promoter (De et al. 2011), into tobacco leaves by *Agrobacterium* infiltration. Via merging the micrographs from each channel, we found that the GFP signals detected in OsLAP6/OsPKS1-GFP overlapped with the RFP signals of ER-marker (Fig. 6c). These results supported our prediction that OsLAP6/OsPKS1 is localized to the ER, and suggested that OsLAP6/OsPKS1 may have a similar role to PKSB/LAP6 in the biosynthesis of sporopollenin.

### OsLAP6/OsPKS1 protein is conserved in land plants

**Fig. 7:**
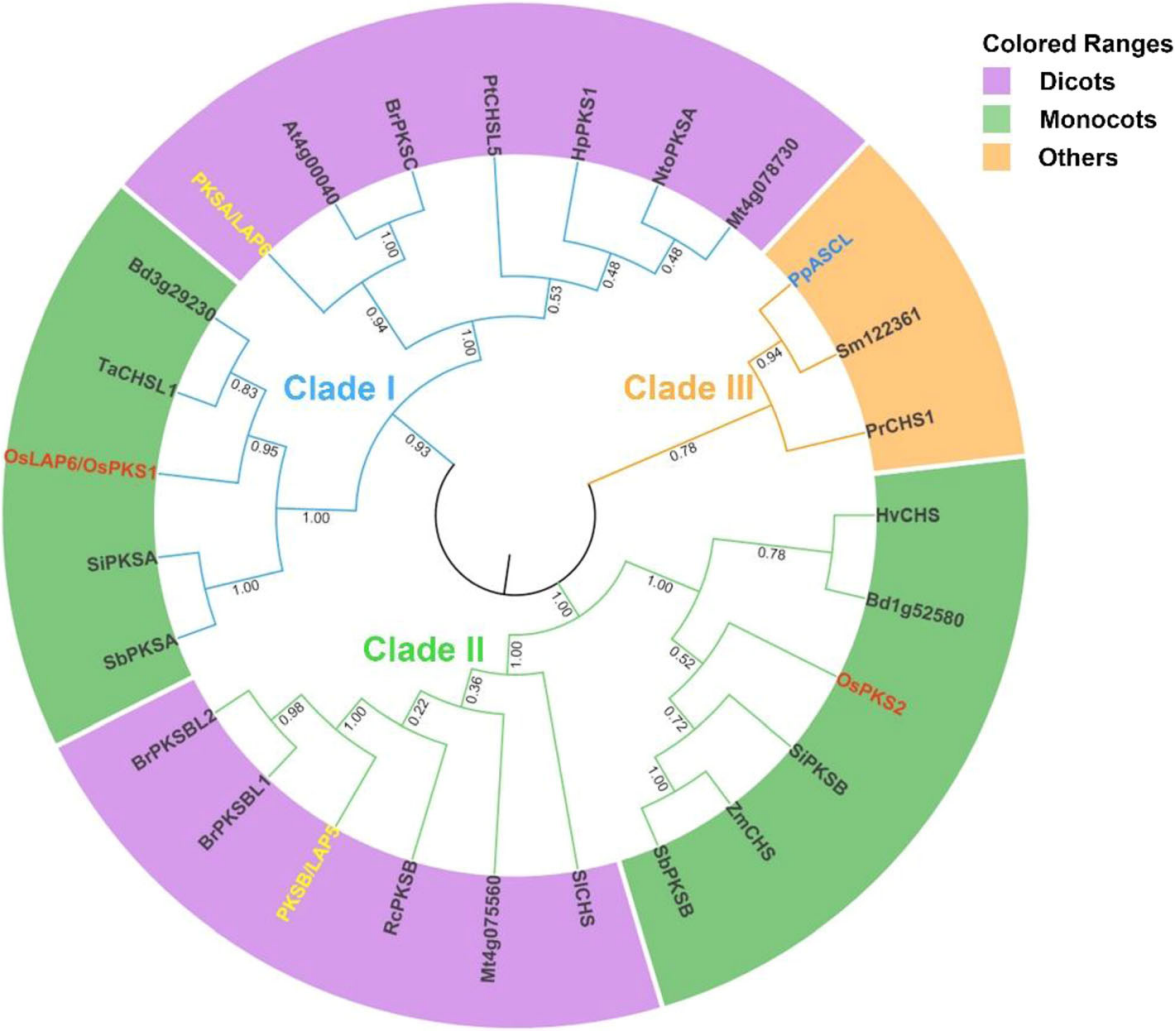
Protein phylogeny analysis among OsLAP6/OsPKS1-related proteins. A Neighbor-Joining phylogeny analyses was performed using MEGA5, based on the alignment results in Additional file 1: Figure S4. The OsLAP6/OsPKS1-related proteins are clearly grouped into three clades. The proteins in clades I and II belong to the homologous proteins of PKSA/LAP6 and PKSB/LAP5 groups, respectively. The numbers at the nodes indicate the bootstrap value. The detailed information of OsLAP6/OsPKS1-related proteins are listed in Additional file 2: Table S2

A previous study reported that OsPKS1 protein shared the similar products of enzymatic reaction with PKSA/LAP6 (Wang et al. 2013), suggesting a conserved biochemical function of PKSs in sporopollenin metabolism between monocots and dicots. In order to gain additional insight of evolutionary and functional conservation among OsLAP6/OsPKS1 and its orthologs in plant species, we used the BLASTP tool in National Center for Biotechnology Information (NCBI, https://www.ncbi.nlm.nih.gov) with the full-length amino acids sequence of OsLAP6/OsPKS1 as a query and retrieved the 26 closest relatives. These 26 OsLAP6/OsPKS1-realtives existed in 19 different plant species from angiosperms, gymnosperms, and cryptogams (Additional file 2: Table S2). Peptide alignment indicated that, including OsLAP6/OsPKS1, all 27 proteins had high conservation of active sites, product-binding sites, and substrate-binding sites (Additional file 1: Figure S4), implying that these functional sites are evolutionarily conserved in land plants. Furthermore, based on the results of protein sequences alignment, we constructed a neighbor-joining phylogenetic tree of these 27 proteins (Fig. 7). The OsLAP6/OsPKS1-realtives were clustered into three clades. Both monocots and dicots plants were grouped into clade I and clade II, and the members in clade III belong to pteridophytes, mosses, and gymnosperms. OsLAP6/OsPKS1 had ~69%, ~54%, and ~53% identity to the proteins in clade I, II, and III, respectively. Besides, because *OsLAP6/OsPKS1* had a strong and specific expression in rice flowers (Fig. 5), we therefore retrieved the electronic fluorescent pictograph (eFP) browser in the Bio-Analytic Resource for Plant Biology (BAR, http://bar.utoronto.ca). We found that several homologous genes of *OsLAP6/OsPKS1* in *Arabidopsis thaliana*, *Brachypodium distachyon*, *Physcomitrella patens*, *Populus trichocarpa*, *Sorghum bicolor*, and *Triticum aestivum* were also predominantly transcribed in their floral organs (data not shown). These results suggested that these genes may have a functional similarity to *OsLAP6/OsPKS1*, and OsLAP6/OsPKS1 protein is conserved in land plants.

## Discussion

Pollen exine is a hard and sticky structure; it plays an important role in protecting pollen from environmental stress, promoting pollen germination, and recognition between pollen and stigma (Blackmore et al. 2007). Thus, the formation of pollen exine is crucial for the development and function of male gametes in flowering plants (Ariizumi and Toriyama 2010). Even though much progress has been made in understanding pollen exine development in *Arabidopsis*, our knowledge of its formation mechanisms in rice is still inadequate. Here, we characterized a complete male sterile mutant, *oslap6*, which has defects in pollen exine formation. MutMap analysis demonstrated that a point mutation of *OsLAP6/OsPKS1* gene caused the mutant phenotype. Loss-of-function of *OsLAP6/OsPKS1* by using CRISPR/Cas9 genomic editing tool also resulted in the same phenotype of aborted pollen grains. These findings suggested that *OsLAP6/OsPKS1* plays an important role in rice male gametes development.

### *OsLAP6/OsPKS1* plays a crucial role in pollen exine formation by regulating bacula elongation

*OsLAP6*, also called *OsPKS1* (Wang et al. 2013), encodes a plant PKS III superfamily protein, which catalyzes the synthesis of various plant secondary metabolites (Xie et al. 2016). Secondary metabolites are involved in regulating a variety of developmental processes in plants, such as disease resistance, environmental stress response, and sexual reproduction (Wink 1988; Kliebenstein 2004; Gershenzon 1984; Galambosi et al. 2009; Theis and Lerdau 2003). In *Arabidopsis* or rice, mutations in PKS genes, such as *PKSA/LAP6*, *PKSB/LAP5*, and *OsPKS2*, led to varying levels of defects in pollen exine (Dobritsa et al. 2010; Kim et al. 2010; Zhu et al. 2017). In *Arabidopsis*, although both single mutants of *pksa/lap6* and *pksb/lap5* were fertile, their double mutants had a male sterile phenotype (Dobritsa et al. 2010; Kim et al. 2010). In addition, the rice *ospks2* mutants also showed male sterility (Zhu et al. 2017). A previous study reported that the insertion of the *Tos17* transposon at the first intron of the *OsPKS1* gene caused the partial-male sterility and reduced seeds rate phenotype in rice (Wang et al. 2013). However, the *oslap6* mutant had a complete male sterile phenotype due to pollen abortion (Fig. 1). Further cytological examination revealed that the microspores of the *oslap6* mutant started to shrink from stage 9, and ultimately formed adhesive and aborted pollen with deformed pollen exine patterning (Figs. 2 and 3). Through molecular detection and co-segregation analysis, we found that a SNP variation in the second exon of *OsLAP6/OSPKS1* gene caused the phenotype of *oslap6* mutant (Fig. 4a and b). These results indicated that *oslap6* is a strong mutant of *OsLAP6/OSPKS1* gene, and suggested a crucial role for *OsLAP6/OsPKS1* in pollen viability and pollen exine formation.

Unlike *Arabidopsis* exine consisting of a thin nexine layer, a longer bacula, and a semi-open tectum layer, the pollen exine in rice has continuous tectum, thick nexine, and high-dense bacula (Zhang et al. 2016; Zhang and Li 2014; Wilson and Zhang 2009). By connecting the tectum layer and the nexine layer, mature bacula raise the discontinuous interspace between these two layers, leading to the formation of a double-layer structure in rice pollen exine; and this bilayer pollen exine structure plays key roles in rice pollen for resisting of abiotic or biotic stress and the maintenance of pollen morphology (Shi et al. 2015; Lu et al. 2002). Previous reports suggested that the bacula of mature rice pollen is formed by the elongation of probacula (Li and Zhang 2010). In *Arabidopsis*, *PKSA/LAP6* is an orthologous gene of *OsLAP6/OsPKS1*; and the exine of less adhesive but fertile pollen grains from *pksa/lap6* mutants was thinner, with shorter bacula (Kim et al. 2010). Our SEM and TEM observation results showed that, at the mature pollen stage, the granular and collapsed bacula in deformed exine of *oslap6* mutants resulted in sterile and aborted pollen (Fig. 3). Therefore, we assumed that the integrity of bacula is important for the viability and fine organization of exine structures in rice pollen, and suggested that *OsLAP6/OsPKS1* may regulate the formation of pollen exine by affecting the elongation of bacula.

### OsLAP6/OsPKS1 may be involved in the conserved biosynthetic process of sporopollenin in land plants

The main component of pollen exine is sporopollenin, which is derived from tapetal cells and deposited on the surface of the microspores (McCormick 2013; Ariizumi and Toriyama 2010). The sporopollenin biosynthetic pathway is conserved in monocots and dicots. (Gomez et al. 2015). In *Arabidopsis*, ACOS5, PKSA/LAP6, and PKSB/LAP5 are immunolocalized together to the ER of the tapetum and form a complex for sporopollenin biosynthesis (de Azevedo Souza et al. 2009; Dobritsa et al. 2010; Kim et al. 2010; Lallemand et al. 2013). In rice, OsACOS12, OsLAP6/OsPKS1, and OsPKS2 are orthologs of ACOS5, PKSA/LAP6, and PKSB/LAP5, respectively. Both *OsACOS12* and *OsPKS2* exhibited a strong expression in tapetum, and the proteins encoded by them have the similar enzymatic function with their *Arabidopsis* orthologs (Li et al. 2016c; Yang et al. 2017; Zou et al. 2017a; Zhu et al. 2017). Our previous work showed that OsACOS12 is partially localized to the ER (Zou et al. 2017a), and we also found preferential localization of OsPKS2 in the ER (unpublished data). Besides, *osacos12* and *ospks2* mutants were male sterile due to the defects in pollen exine formation (Li et al. 2016c; Yang et al. 2017; Zou et al. 2017a; Zhu et al. 2017). Likewise, our expression analysis and subcellular location assay indicated that OsLAP6/OsPKS1 has a tapetum-specific expression pattern and is localized to the ER (Figs. 5 and 6), and cytological observation revealed the defective exine formation in *oslap6* mutants (Figs. 2 and 3). Simultaneously, previous study showed that OsLAP6/OsPKS1 shared the similar products of enzymatic reaction with PKSA/LAP6 (Wang et al. 2013). These results suggested that, consistent with OsACOS12 and OsPKS2, OsLAP6/OsPKS1 might share the same role with its *Arabidopsis* ortholog in sporopollenin metabolic process.

In addition, tobacco NtPKS1 shared 79% identity with PKSA/LAP6, and was found to be expressed in tapetum of *Nicotiana sylvestris* (Atanassov et al. 1998). RNA interference mutants of *NtPKS1* was male sterile with disorganized pollen exine (Wang et al. 2013). Similarly, *PpASCL*, the moss orthologous gene of *PKSA/LAP6*, encodes a sporophyte-specific enzyme that exhibits analogous catalytic activity to which of PKSA/LAP6 in vitro (Colpitts et al. 2011). Due to the sporopollenin biosynthetic defects, knockout mutants of *PpASCL* produce nonviable and deformed spores (Daku et al. 2016), suggesting a conservation of sporopollenin biosynthesis in land plants. Our peptides alignment and phylogenetic analysis indicated that the OsLAP6/OsPKS1 protein is conserved among land plants. These findings suggested that OsLAP6/OsPKS1 might participate in the conserved sporopollenin metabolism in land plants.

### Manipulation of *OsLAP6/OsPKS1* has implication in hybrid rice breeding

By using the CRISPR/Cas9 genomic editing tool to generate the insertion or deletion mutations, we knocked out the *OsLAP6/OsPKS1* gene and obtained many its loss-of-function mutants. All of these mutants are in *japonica* background (Nipponbare) and have normal vegetative growth and male sterile phenotypes (Fig. 4), which was in agreement with the *oslap6* mutants in *indica* background (9311, Fig. 1). These results suggest that *OsLAP6/OsPKS1* is a key molecular switch of the male fertility in both *japonica* and *indica* rice.

Rice, as one of the most important crops for nutrition and calorie intake of human, feeds nearly half of the world’s population (Virmani 1994; Cheng et al. 2007; Khush 2013). The improvement of rice yield requires the hybrid breeding that depends on male sterile lines. Presently, the main production methods of commercial hybrid rice are three-line system and two-line system, which are based on cytoplasmic male sterile (CMS) lines and photoperiod/thermo-sensitive genic male sterile (PTGMS) lines, respectively (Joshi et al. 2001; Li et al. 2012; Fan et al. 2017; Chang et al. 2016; Li et al. 2016a). However, in three-line system, the germplasm resources of the restorer lines are narrow, and the genetic diversity between CMS and restorer lines is deficient (Cai et al. 2001; Michel et al. 2009; Chang et al. 2016). Thus, the pyramiding of various outstanding traits to obtain excellent hybrid rice varieties is a difficulty in the three-line system. On the other hand, although the PTGMS line-based two-line system breaks the restrictions of the restorer lines and the maintainer lines, which greatly improving the freedom of parent selection (Lopez and Virmani 2000; Gnanasekaran and Vivekanandan 2008), the male fertility of PTGMS lines are sensitive to the environmental changes (Li et al. 2016a; Chen et al. 2010). This problem causes self-pollination and reduces the purity of the hybrid seed, and leads to the failure of large-scale seeds production. However, the latest genomic editing tool and transgenic technology provides the possible advances in rice heterosis utilization. The combined use of genetic engineering, such as genomic site-directed mutagenesis method and Seed Production Technology (SPT), has gradually become an effective way to create male sterile lines for pollinating-crops (Quanlin et al. 2016; Zhou et al. 2016; Zhang et al. 2017; Wu et al. 2016; Chang et al. 2016). In general, we can use the CRISPR/CAS9 system to knock out rice male fertility controlling genes to obtain genic male sterile (GMS) lines; then introduce the SPT constructs, which containing closely linked pollen fertility restoring genes, pollen lethal genes, and screening marker genes, into GMS lines to obtain the corresponding maintainer lines. In this strategy, we can theoretically develop any excellent rice germplasm resource into commercial GMS line by manipulation of its male fertility genes. In the present study, through the genetic and molecular biological analysis, we demonstrated that *OsLAP6/OsPKS1* is a key regulatory gene of male fertility in both *japonica* and *indica* rice, and thus proposed that manipulation of *OsLAP6/OsPKS1* in this strategy has great potential for applications in hybrid rice breeding.

## Conclusions

The *oslap6* mutant produced aborted pollen and has a complete male sterile phenotype that is caused by defective pollen exine formation. MutMap and co-segregation analysis indicated that a SNP variation in an orthologous gene of *Arabidopsis PKSA/LAP6*, *OsLAP6/OsPKS1*, resulted in the phenotype of male sterility. Loss-of-function mutants of *OsLAP6/OsPKS1* were also completely male sterile. *OsLAP6/OsPKS1* has a tapetum-specific expression pattern, and encodes an ER-localized protein that showed the functional conservation of sporopollenin metabolic process in land plants. In summary, our results suggest that, as an essential manipulator of rice male fertility, *OsLAP6/OsPKS1* may be involved in a conserved sporopollenin precursor biosynthetic pathway in land plants, and has possible applications in hybrid rice breeding.

## Methods

### Plant materials and growth conditions

The *oslpa6* mutant line was identified from an ethyl methanesulfonate (EMS)-induced mutant library of an *indica* cultivar 9311. EMS treatment and mutant screening was performed as described previously (Abe et al. 2012; Rao 1977). All plants were grown in paddies at the Rice Research Institute of Sichuan Agricultural University (Chengdu, China) and Hainan (Lingshui, China) under normal cultivation conditions.

### Phenotypic characterization of the *oslap6* mutant

The phenotypes of the whole plants and floral organs were photographed with a Canon EOS 1200D digital camera. Phenotypic observations of semi-thin sections and Scanning Electronic Microscopy (SEM) were carried out as previously described (Zou et al. 2017b). Transmission electron microscopy (TEM) was performed as described in a previous study (Qin et al. 2013). The anthers from different developmental stages, as defined by Zhang and Wilson (2009) and Zhang et al. (2011), were collected based on spikelet length and lemma/palea morphology.

### Gene mapping and phenotype association assay

The *oslap6* mutant was backcrossed with the wild type (9311), and the resulting F1 plant was further selfed to generate the F2 population. Fifty male sterile plants from the F2 population were randomly selected for DNA extraction, and equal amounts of DNA were pooled and sequenced. The SNP indexes were calculated as the MutMap method described previously (Abe et al. 2012). To verify the association of the candidate mutation in *LOC_Os10g34360* and the male sterile phenotype of *oslap6*, 48 fertile plants and 12 male sterile plants in the F2 population were further genotyped by using direct sequencing of the PCR products that amplified by primer set OsLAP6-SEQ1. All the primers used in this study are listed in Additional file 2: Table S3.

### CRISPR/Cas9 plasmids construction, plant transformation and mutation detection

To obtain loss-of-function mutants, we designed two independent target sites at the first and second exon of *LOC_Os10g34360*, respectively (Fig. 4c and h). By using primer sets OsLAP6-KO1 and OsLAP6-KO2, the CRISPR/Cas9 plasmids were generated and introduced into *Agrobacterium tumefaciens* strain EHA105, and transformation of Nipponbare was performed as described previously (Zou et al. 2017b). We also observed the target site sequences of all the CRISPR/Cas9-mediated transgenic plants via direct or cloned sequencing of the PCR products, which were amplified using primer set OsLAP6-SEQ2, and further confirmed the co-segregation of the target mutations and male sterile phenotype of mutants (Additional file 2: Table S2).

### Quantitative real-time PCR and RNA in situ hybridization

Total RNA from various rice tissues was extracted by using RNeasy Plant Mini Kit (Qiagen, Germany) and was reverse transcribed by using the SuperScript™ First-Strand Synthesis System (Life Technologies, USA). Quantitative real-time PCR (qPCR) was performed with primer set OsLAP6-QPCR and soAdvanced™ SYBR Green Supermix (Bio-Rad, USA) by using the CFX96 Real-Time PCR System (Bio-Rad, USA) according to the manufacturer’s instructions. OsACTIN was used as the internal standard gene to normalize the cDNA level of target gene. Three replicates were used in each sample. The relative expression levels were measured as described previously (Chen et al. 2016).

RNA in situ hybridization was conducted as described previously (Li et al. 2016b). An 85 bp and a 104 bp cDNA fragment of *OsLAP6/OsPKS1* were amplified using specific primers (Additional file 2: Table S3), and were then mixed for preparing sense and antisense probes, respectively.

### Histochemical activity assay of GUS

To visualize the expression of *OsLAP6/OsPKS1* gene, a 2.3-kb DNA fragment of *OsLAP6/OsPKS1* promoter (upstream of the start codon ATG) was amplified from WT using primer set OsLAP6-PRO and used to construct the OsLAP6/OsPKS1_pro_::GUS plasmid as described previously (Zou et al. 2017b). The generation of transgenic plants and the histochemical activity detection of GUS in transgenic plants were performed according to the method described previously (Zou et al. 2017a).

### Protein localization of OsLAP6

To detect whether the OsLAP6/OsPKS1 protein is also specifically expressed in tapetum, the promoter region described above and the full-length cDNA of *OsLAP6/OsPKS1* gene were subsequently cloned into the expression vector pA7-GFP to generate the OsLAP6/OsPKS1_pro_::OsLAP6/OsPKS1-GFP construct. The transgenic plants were generated as described previously (Aldemita and Hodges 1996). The OsLAP6/OsPKS1-GFP florescence was observed with a confocal laser scanning microscope (Nikon A1, Kanagawa, Japan).

To determine the subcellular localization of OsLAP6/OsPKS1, we replaced the promoter in OsLAP6/OsPKS1pro::OsLAP6/OsPKS1-GFP with the double 35 s promoter, and generated the 2×35S::OsLAP6/OsPKS1-GFP plasmid. The vectors 2×35S::OsLAP6-GFP and ER-marker, a RFP that fused with the KDEL ER-retention signal and also driven by the double 35S promoter (De et al. 2011) were co-transfected into in tobacco (*Nicotiana benthamiana*) leaf epidermal cells, and the signals of GFP and RFP were observed as described previously (Zou et al. 2017b).

### Phylogenetic analysis

The OsLAP6/OsPKS1-related proteins in 19 plant species (Fig. 7 and Additional file 2: Table S2) were identified by NCBI BLASTP (https://blast.ncbi.nlm.nih.gov/Blast.cgi) with default parameters by using the full-length peptides sequence of OsLAP6/OsPKS1 as a query, and the sequences retrieved were aligned with ClustalW (Goujon et al. 2010). The MEGA5 program using the Neighbor-Joining method with default parameters besides 1000 bootstrap replications was used to generate the phylogenetic tree (Tamura et al. 2011), which was further edited by using the Interactive Tree of Life on line tool (iTOL, http://itol.embl.de) (Letunic and Bork 2016).

## Additional files

Additional file 1: Figure S1. Distributions of SNP index along chromosomes of *oslap6* mutant. **Figure S2.** Sequence analysis of *oslap6* and loss-of-function mutants of *OsLAP6/OSPKS1.* **Figure S3.** Protein sequence alignment between PKSA/LAP6 and OsLAP6/OsPKS1. **Figure S4.** Peptides alignment of OsLAP6/OsPKS1-related proteins. (DOCX 2692 kb)

Additional file 2: Table S1. Phenotype and genotype association analyses in BCF2 plants of loss-of-function mutants (generated by using CRISPR/Cas9 genomic editing tool). **Table S2.** The information of OsLAP6/OsPKS1-related proteins. **Table S3.** Primers used in this study. (DOCX 22 kb)
