# Supplementary material for: OsLAP6/OsPKS1, an orthologue of Arabidopsis PKSA/LAP6, is critical for proper pollen exine formation
Source: Rice (N Y). 2017 Dec 28;10:53. doi: 10.1186/s12284-017-0191-0 (PMC5745217; doi:10.1186/s12284-017-0191-0)
Supplement: Supplementary file 1 — Distributions of SNP index along chromosomes of oslap6 mutant. Figure S2. Sequence analysis of oslap6 and loss-of-function mutants of OsLAP6/OSPKS1. Figure S3. Protein sequence alignment between PKSA/LAP6 and OsLAP6/OsPKS1. Figure S4. Peptides alignment of OsLAP6/OsPKS1-related proteins. (DOCX 2692 kb) [file 12284_2017_191_MOESM1_ESM.docx]

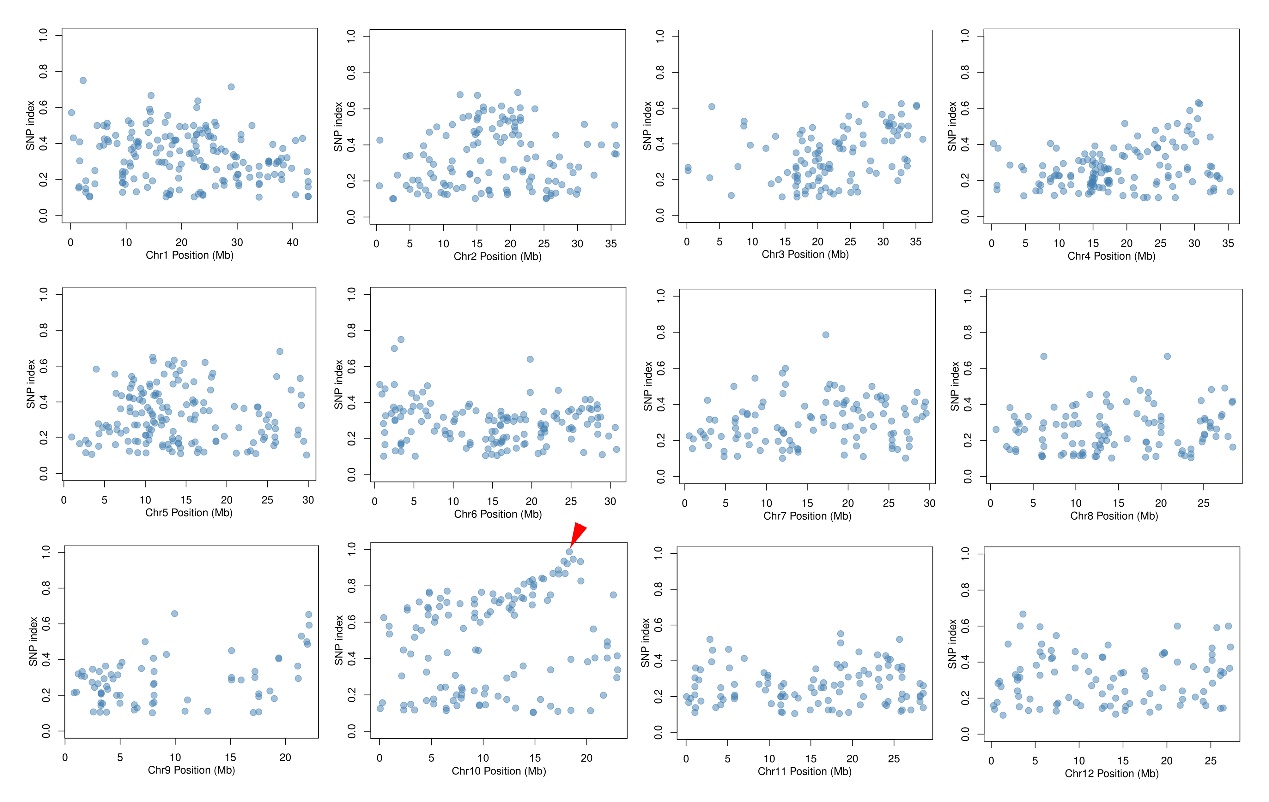


**Additional file 1 Figure S1.** **Distributions of SNP index along chromosomes of *oslap6* mutant.** The candidate region harboring causal mutation is located between 17.79 Mbp and 18.69 Mbp on chromosome 10 and indicated by a red arrow.


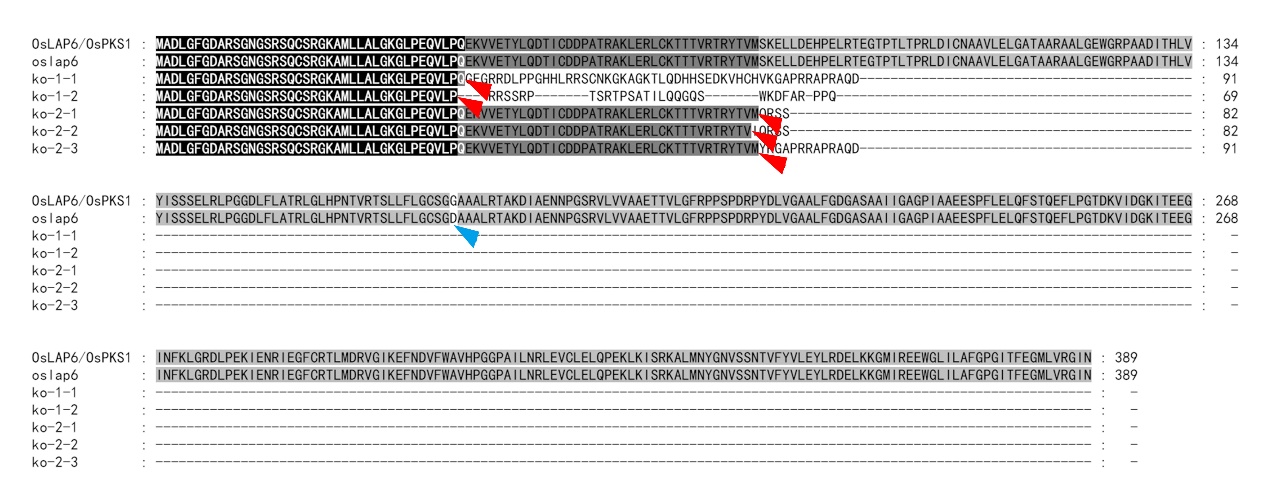


**Additional file 1 Figure S2. Sequence analysis of *oslap6* and loss-of-function mutants of *OsLAP6/OSPKS1*.** Putative amino acid sequences alignment of mutations as de-scribed in Fig. 4c and h. The sequences were aligned by using ClustalW, and displayed by using BOXSHADE. The red arrows and blue arrow indicate the truncated amino acids of loss-of-function mutants and amino acid substitution in *oslap6* mutants, respectively.


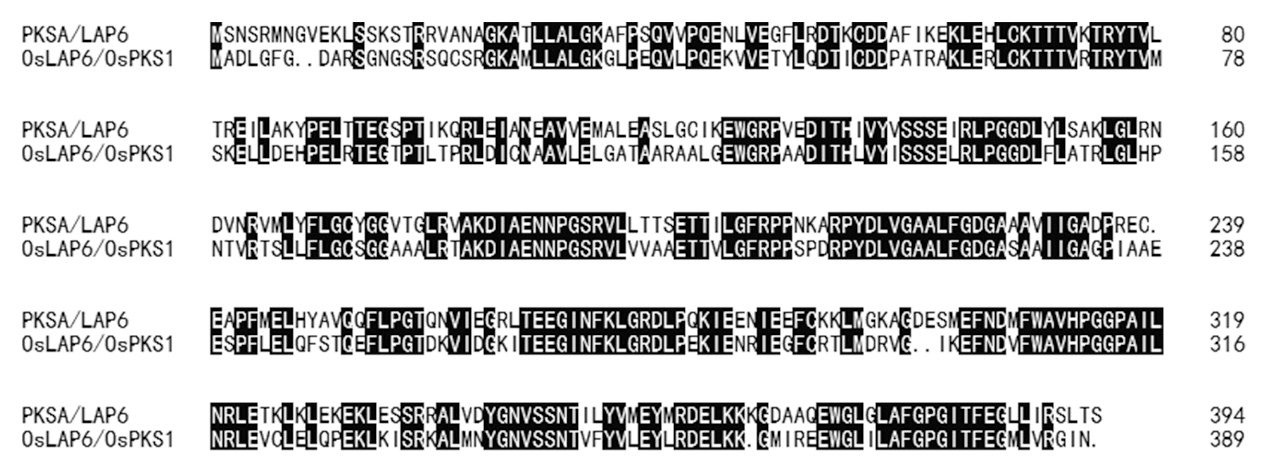


**Additional file 1 Figure S3. Protein sequence alignment between PKSA/LAP6 and OsLAP6/OsPKS1.** The sequences were aligned by using DNA-MAN, and displayed by using BOXSHADE.


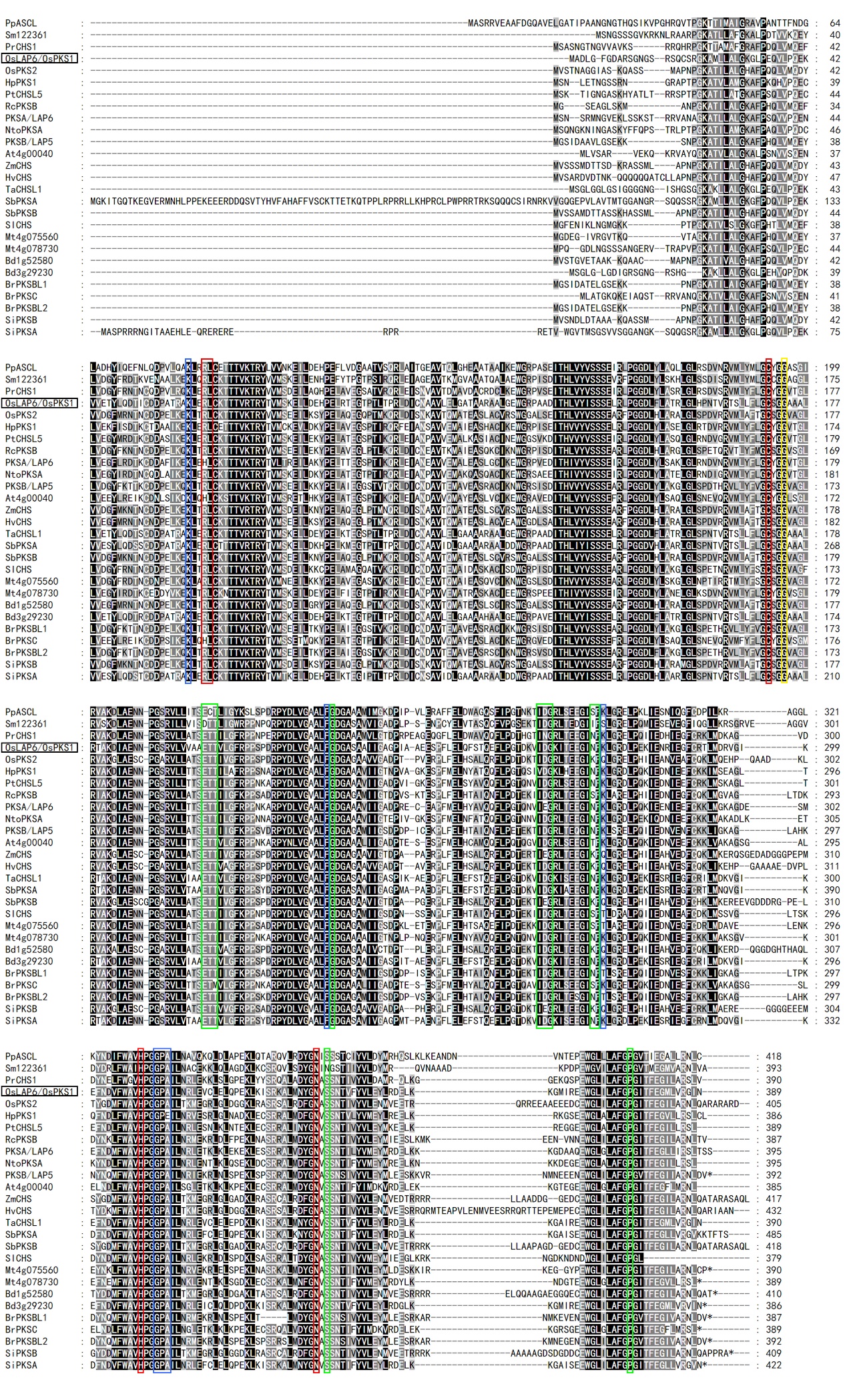


**Additional file 1 Figure S4. Peptides alignment of OsLAP6/OsPKS1-related proteins.** The sequences were aligned by using ClustalW, and displayed by using BOXSHADE. The red, green, and blue frames indicate the conserved active sites, product-binding sites, and substrate-binding sites in OsLAP6/OsPKS1-related proteins, respectively. The yellow frame showed that the amino acid substitution site caused by a SNP mutation of *OsLAP6/OsPKS1* gene in *oslap6* mutants is also conserved in OsLAP6/OsPKS1-related proteins.
