# Supplementary material for: OsLAP6/OsPKS1, an orthologue of Arabidopsis PKSA/LAP6, is critical for proper pollen exine formation
Source: Rice (N Y). 2017 Dec 28;10:53. doi: 10.1186/s12284-017-0191-0 (PMC5745217; doi:10.1186/s12284-017-0191-0)
Supplement: Supplementary file 2 — Phenotype and genotype association analyses in BCF2 plants of loss-of-function mutants (generated by using CRISPR/Cas9 genomic editing tool). Table S2. The information of OsLAP6/OsPKS1-related proteins. Table S3. Primers used in this study. (DOCX 22 kb) [file 12284_2017_191_MOESM2_ESM.docx]

**Additional file 2 Table S1. Phenotype and genotype association analyses in BCF2 plants of loss-of-function mutants (generated by using CRISPR/Cas9 genomic editing tool).**

| Line | Fertility | Homo-WT (+/+) | Heterozygous (+/-) | Biallelic or Homo-MT (-/-) |
| --- | --- | --- | --- | --- |
| *ko-1-1* | Normal fertility | 5 | 6 | 0 |
|  | Male sterility | 0 | 0 | 25 |
| *ko-1-2* | Normal fertility | 3 | 7 | 0 |
|  | Male sterility | 0 | 0 | 22 |
| *ko-2-1* | Normal fertility | 6 | 10 | 0 |
|  | Male sterility | 0 | 0 | 15 |
| *ko-2-2* | Normal fertility | 3 | 6 | 0 |
|  | Male sterility | 0 | 0 | 18 |
| *ko-2-3* | Normal fertility | 5 | 7 | 0 |
|  | Male sterility | 0 | 0 | 19 |

All these plants are in *japonica* Nipponbare background. The numbers in this table indicate the numbers of different genotype plants in different phenotypes.

**Additional file 2 Table S2. The information of OsLAP6/OsPKS1-related proteins.**

| Proteins | GenBank Acessions | Species | Identity (%)* |
| --- | --- | --- | --- |
| At4g00040 | NP_191915 | *Arabidopsis thaliana* | 58 |
| Bd1g52580 | KQK20123 | *Brachypodium distachyon* | 51 |
| Bd3g29230 | KQJ97175 | *Brachypodium distachyon* | 90 |
| BrPKSBL1 | XP_009108194 | *Brassica rapa* | 56 |
| BrPKSBL2 | XP_009138330 | *Brassica rapa* | 56 |
| BrPKSC | XP_013626817 | *Brassica rapa* | 58 |
| HpPKS1 | JQ073294 | *Hypericum perforatum* | 60 |
| HvCHS | AAV49989 | *Hordeum vulgare* | 43 |
| Mt4g075560 | KEH30597 | *Medicago truncatula* | 59 |
| Mt4g078730 | AES89695 | *Medicago truncatula* | 62 |
| NtoPKSA | XP_009618741 | *Nicotiana tomentosiformis* | 63 |
| OsLAP6/OsPKS1 | NP_001064891 | *Oryza sativa* | 100 |
| OsPKS2 | NP_001059449 | *Oryza sativa* | 53 |
| PKSA/LAP6 | NP_171707 | *Arabidopsis thaliana* | 61 |
| PKSB/LAP5 | NP_567971 | *Arabidopsis thaliana* | 57 |
| PpASCL | XP_001781520 | *Physcomitrella patens* | 49 |
| PrCHS1 | AAB80804 | *Pinus radiata* | 58 |
| PtCHSL5 | XP_002326830 | *Populus trichocarpa* | 62 |
| RcPKSB | XP_002529257 | *Ricinus communis* | 59 |
| SbPKSA | XP_002467058 | *Sorghum bicolor* | 69 |
| SbPKSB | XP_002461886 | *Sorghum bicolor* | 50 |
| SiPKSA | XP_004982809 | *Setaria italica* | 80 |
| SiPKSB | XP_004956033 | *Setaria italica* | 52 |
| SlCHS | BAE80096 | *Silene latifolia* | 55 |
| Sm122361 | XP_002985571 | *Selaginella moellendorffii* | 51 |
| TaCHSL1 | EU408770 | *Triticum aestivum* | 91 |
| ZmCHS | NP_001149508 | *Zea mays* | 50 |

* The percentage identities represent the similarity of two sequences between corresponding proteins and OsLAP6/OsPKS1.

**Additional file 2 Table S3: Primers used in this study**

| Primer ID | Sequence(5’ to 3’) | Purpose |
| --- | --- | --- |
| OsLAP6-QPCR-F | AAGGGCAAAGCTGGAAAGA | qPCR analyses |
| OsLAP6-QPCR-R | CTCACTGGACGAGATGTAGACAA |  |
| OsACTIN-QPCR-F | GCTATGTACGTCGCCATCCA | qPCR analyses |
| OsACTIN-QPCR-R | GGACAGTGTGGCTGACACCAT |  |
| OsLAP6-PRO-F | aagcttTGAGGTCGGCGGCGAT | GUS assay and tissue-specific localization of OsLAP6/OsPKS1 |
| OsLAP6-PRO-R | ggatccCTTCGCCACCCGGTATCCT |  |
| OsLAP6-YFP-F | aagcttATGGCTGACCTTGGATTC | Tissue-specific and subcellular localization of OsLAP6/OsPKS1 |
| OsLAP6-YFP-R | ggatccGTTAATGCCTCGAACTATCA |  |
| OsLAP6-SEQ1-F | TGTCTACATCTCGTCCAGTG | Genotype and phenotype association analysis |
| OsLAP6-SEQ1-R | TCTTGCCATCAATTACCTT |  |
| OsLAP6-SEQ2-F | CTACTGAGGCGAAAGGATA | Genotype and phenotype association analysis |
| OsLAP6-SEQ2-R | ACTGGACGAGATGTAGACAA |  |
| OsLAP6-KO1-F | ggcaGCAAGTTCTTCCCCAGGAGA | CRISPR/Cas9 genomic editing |
| OsLAP6-KO1-R | aaacTCTCCTGGGGAAGAACTTGC |  |
| OsLAP6-KO2 -F | ggcaGAGGTACACTGTCATGTCAA | CRISPR/Cas9 genomic editing |
| OsLAP6-KO2-R | aaacTTGACATGACAGTGTACCTC |  |
| OsLAP6-AS-F | TCCATGAGTGTCCTGCAGA | Antisense probe |
| OsLAP6-AS-R | ATTGATGGCAAGATCACTG |  |
| OsLAP6-S-F | AGCAGGCCCCATTGCTGCT | Sense probe |
| OsLAP6-S-R | TACCTTGTCCGTCCCTGGT |  |

The construction adapter is in lower character.
